# Veterinarian Nominated Common Conditions of Rabbits and Guinea Pigs Compared with Published Literature

**DOI:** 10.3390/vetsci4040058

**Published:** 2017-11-22

**Authors:** Natalie J. Robinson, Emma Lyons, Douglas Grindlay, Marnie L. Brennan

**Affiliations:** 1 Centre for Evidence-Based Veterinary Medicine, School of Veterinary Medicine and Science, University of Nottingham, Sutton Bonington Campus, Loughborough LE12 5RD, UK; evg21@hotmail.co.uk (E.L.); marnie.brennan@nottingham.ac.uk (M.L.B.); 2 Centre for Evidence-based Dermatology, University of Nottingham, Kings Meadow Campus, Nottingham NG7 2NR, UK; douglas.grindlay@nottingham.ac.uk

**Keywords:** rabbit, guinea pig, veterinary literature, common conditions, survey

## Abstract

Rabbits and guinea pigs are increasingly popular pets in the UK, yet little is known about their common ailments, or how these relate to what appears in the published literature. The aim of this study was to characterise the common conditions of rabbits and guinea pigs, and to compare these with the topics found in the published literature. Information about the common conditions seen in rabbits and guinea pigs in clinical practice was obtained from a survey of UK veterinarians. The common conditions seen were compared with results from a structured literature search. Conditions relating to the dental (29.9%), and skin (37.6%) body systems were commonly nominated by veterinarians for rabbits and guinea pigs, respectively. A total of 655 rabbit and 1086 guinea pig citations were examined and there appeared to be a mismatch between the conditions nominated in the veterinary questionnaire, and those found in the literature. This is the first time that the published literature has been compared to the nominated caseload of veterinarians in practice, and there is concern that the literature about rabbits and guinea pigs may not be representative of, or relevant to the caseload seen in clinical practice. This is of importance for clinicians being able to apply an objective, evidence-based approach. The publishing of clinically-relevant, research-based evidence should be prioritised.

## 1. Introduction

Rabbits and guinea pigs are increasingly popular pets, with an estimated 1.1 million pet rabbits [1] and 0.5 million pet guinea pigs [2] in the United Kingdom (UK). Rabbits and guinea pigs are also now the third and fourth most frequently presented small animal species seen by UK veterinary practices [3], however, veterinarians feel there is less information available on these species compared with cats and dogs [4]. This may be due to a lack of relevant evidence, or poor awareness of, or access to, existing evidence.

In order for evidence-based medicine to progress, clinical questions relevant to veterinary practice need to be identified. Examining the veterinary caseload, in conjunction with the existing evidence base, will help pinpoint questions relevant to practice and highlight where current knowledge gaps exist. This will enable relevant topics for future research to be prioritised and veterinary curricula modified to reflect common conditions seen in clinical practice. In addition, this will enable identification of ways in which awareness of and access to the existing literature could be improved. Recent studies have examined the small animal caseload in order to identify common conditions, however, there has often been a focus primarily on cats and dogs [5,6,7]. Only a few studies have attempted to examine which conditions most commonly affect rabbits and guinea pigs, although there is uncertainty as to how these relate to the vet visiting population in the UK [8,9,10,11]. It is additionally unclear how much veterinary literature is currently available on each of the conditions previously identified.

The aim of this study was to characterise the common conditions of rabbits and guinea pigs as nominated by UK veterinarians, as well as the amount of information veterinarians perceive to be available for each of these conditions. A second aim was to determine the amount and type of indexed veterinary literature available for each of these species, and to compare this with the perceived amount of information available.

## 2. Materials and Methods

A questionnaire was developed by the Centre for Evidence-based Veterinary Medicine (CEVM) aimed at a target population of all practising UK veterinary surgeons. The questionnaire contained 36 questions divided into four sections covering: respondent demographics; common species and conditions encountered in practice; evidence-based veterinary medicine; sources of information or evidence used by the respondents (a copy of the questionnaire is available upon request). Results relating to three sections of the questionnaire have previously been reported [4,12,13]. Only the second section of the questionnaire, which included questions about common species and conditions, will be considered here. Participants who currently carried out some clinical work were asked to nominate up to four species they frequently encountered in practice and to list the three main conditions or complaints they saw in each species. For each of these conditions, respondents were required to rate how much information they perceived was available on them from “none”, “a little”, “some”, “a lot”, or “don’t know”. The questionnaire was developed using Teleform V.10.5.2 (Verity Inc., Santa Clara, CA, United States, 2010), a program which enables efficient processing and coding of questionnaires. Initial pre-testing of the survey was carried out within the CEVM, and three pilot studies were subsequently carried out (24, 25, and eight participants, respectively).

The questionnaire was distributed to a list of all members of the Royal College of Veterinary Surgeons (RCVS) who had consented to be contacted by external organisations for research and marketing purposes. Participants had the option to complete either the paper questionnaire or an online questionnaire. Reminders were sent to non-responders six and 10 weeks after the initial questionnaire. Further details about questionnaire development, piloting, and distribution have been published previously [4].

All rabbit and guinea pig data from the questionnaire were compiled into a Microsoft Excel table. The specific conditions or complaints listed and the responses regarding the amount of information available (henceforth referred to as “information quantity rating”) were examined. The conditions or complaints were categorised according to body system affected and the categories used were as follows: behaviour; cardiology; dental; endocrinology; gastrointestinal; musculoskeletal; neurological; non-specific; ophthalmology; preventive medicine; reproduction; respiratory; skin; and urinary/renal (see Supplementary Materials for definitions).

Two literature searches, one for each species, were conducted in CAB Abstracts 1910 to 2014 using the OVID interface in November 2014. This database was used as previous research has suggested it provides the best coverage of veterinary journals [14] and in addition, contains a subset of veterinary citations, allowing searching of these citations specifically. The searches were structured to include only species subject headings (not free text terms), disease/veterinary medicine subject headings and were confined to the veterinary subset (ve.ss). This approach was used to optimise the number of relevant citations returned as in-depth analysis of all citations was not carried out. The rabbit search strategy conducted was as follows:

(exp rabbits/OR exp Oryctolagus/) AND (exp rabbit diseases OR exp diagnosis/OR exp diagnostic techniques/OR exp surgery/OR exp surgical operations/OR exp drug therapy/OR exp treatment/OR exp therapy/OR exp intervention/OR exp prophylaxis OR exp prevention/OR exp preventive medicine/OR exp disease control/OR exp veterinary medicine/OR exp veterinary practice/) AND ve.ss.

The guinea pig search strategy conducted was as follows:

(exp guineapigs/OR exp Cavia/) AND (exp diagnosis/OR exp diagnostic techniques/OR exp surgery/OR exp surgical operations/OR exp drug therapy/OR exp treatment/OR exp therapy/OR exp intervention/OR exp prophylaxis OR exp prevention/OR exp preventive medicine/OR exp disease control/OR exp veterinary medicine/OR exp veterinary practice/) AND ve.ss.

All searches were conducted by one author (E.L.) who also completed initial categorisation of all citations, with some later additional categorisation of citations by another author (N.J.R). The earliest citations identified for either species at the time of searching were citations about rabbits from 1972. Due to time constraints, it was not possible to categorise all rabbit citations. Based on the time available, and the time taken to analyse citations from the pilot study (see below), the decision was made to analyse the citations about rabbits from every eighth year, e.g., 1972, 1980, 1988, and so on, until 2012.

An initial screen of citations was conducted prior to categorising each citation. Citations which were not in English, as well as citations which were deemed irrelevant as they did not mention rabbits or guinea pigs in the title or abstract, were removed without further categorisation. Remaining citations from both searches were classified based on body system affected, using the same categories as for the questionnaire (see Supplementary Materials). Each citation was also categorised by type of citation (i.e., whether it was a primary research citation, which was any citation reporting a study belonging to one of the categories listed by CEVM [15], or an expert opinion citation; see Supplementary Materials), and the environment from which the study population came or audience for which the citation was intended (i.e., ‘laboratory’, ‘farm’ or ‘pet’; see Supplementary Materials). Data were also gathered on the year and in which publication the citations appeared.

A small pilot study was conducted in which one author (E.L.) categorised the citations on rabbits from 1972–1974 in order to check the feasibility of the categories used. Ambiguous citations were discussed with two authors (M.L.B. and N.J.R.) to determine how they should be categorised. For example, one citation entitled ‘Treatment for liver lobe torsion in domestic rabbits’ was categorised as:

- Body system/clinical sign: gastrointestinal
- Type of study: opinion
- Environment: pet

The initial dataset from the questionnaire was transferred to Microsoft Excel V.14.0.6 (Microsoft Corporation, Redmond, WA, USA, 2010) for data management. Data relating to rabbits and guinea pigs was identified from this dataset and compiled into a separate Microsoft Excel spreadsheet for each species. Search results from CAB Abstracts were also transferred into Microsoft Excel for data management and categorisation of each citation by body system, type of citation, environment, year of publication, and name of publication. Pivot tables in Microsoft Excel were used to conduct descriptive statistics on data from both the questionnaire and literature search.

## 3. Results

### 3.1. Questionnaire

Of 14,532 questionnaires distributed, 5407 (37.2%) were returned. After excluding questionnaires returned to sender or where the veterinarian was retired or deceased, this left 4842/5407 (89.6%) responses which could be used in the analysis. Of these, 3982 (82.2%) did some clinical practice and noted down the specific conditions or complaints they saw commonly. Rabbits were listed as a commonly seen species by 2170 (54.5%) respondents, and guinea pigs by 682 (17.1%) respondents. As each respondent could list up to three conditions that they saw commonly per species, 6410 instances of rabbit conditions were noted, and 1974 for guinea pigs.

#### 3.1.1. Body System

‘Dental’ (1918/6410; 29.9%) was the most frequently occurring body system category for rabbits, followed by ‘skin’ (1619/6410; 25.3%), then ‘gastrointestinal’ (974/6410; 15.2%) (Table 1). No conditions or complaints were categorised as belonging to the ‘cardiology’ or ‘endocrinology’ body systems.

‘Skin’ (743/1974; 37.6%) was the most frequently occurring body system category for guinea pigs, followed by ‘non-specific’ (365/1974; 18.5%), then ‘dental’ (346/1974; 17.5%) (Table 1). The least frequently used body system categories for guinea pigs were ‘behaviour’ (2/1974; 0.1%) and ‘cardiology’ (1/1974; 0.1%).

#### 3.1.2. Information Quantity Rating

**Figure 1:**
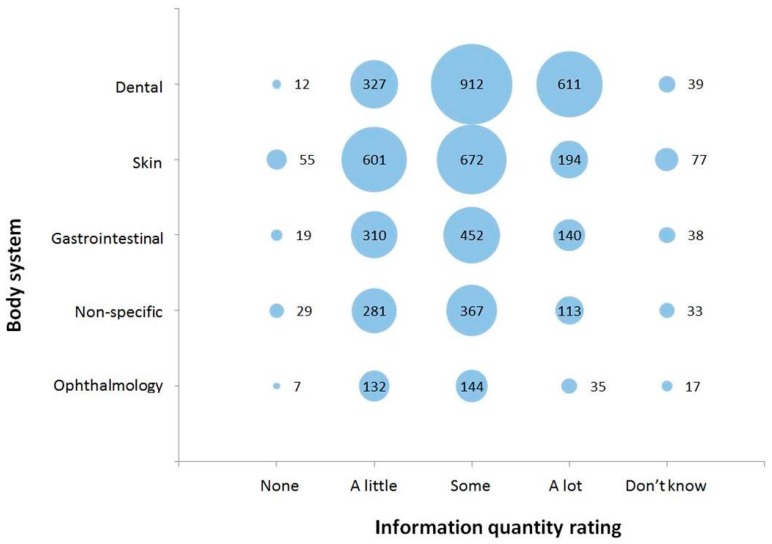
Information quantity rating selected for conditions or complaints affecting the five most commonly categorised body systems in rabbits (*n* = 6324) from a survey of UK veterinarians. Size of the bubble, and the data label within or to the right of each bubble, corresponds to the total number of conditions or complaints fitting into each combination of body system category and information quantity rating.

Data on information quantity rating was complete for 98.7% (6324/6410) of conditions or complaints listed for rabbits (for full details see Table S1, Supplementary Materials). The overall results show that veterinarians perceived there to be ‘a lot’ of information available for only 18.6% (1176/6410) of conditions or complaints they listed. ‘Some’ information was perceived to be available for 45.1% (2855/6324) of the conditions or complaints listed, while ‘a little’ information was perceived to be available for 30.3% (1917/6324) of conditions or complaints listed. Only 2.2% (142/6324) of conditions or complaints listed were perceived as having no information available, while a response of ‘don’t know’ was listed for 3.7% (234/6324) of conditions and complaints. The information quantity rating for the five most commonly categorised body systems is shown in Figure 1. For most body systems, ‘a little’ and ‘some’ were the most frequently selected information quantity ratings. However for conditions or complaints categorised as ‘dental’, ‘some’ or ‘a lot’ were the most frequently selected information quantity ratings.

**Figure 2:**
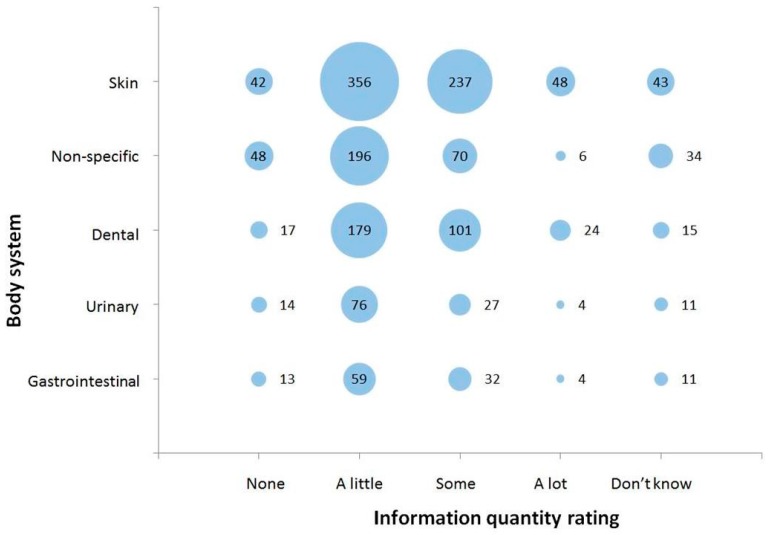
Information quantity rating selected for conditions or complaints affecting the five most commonly categorised body systems in guinea pigs (*n* = 1925) from a survey of UK veterinarians. Size of the bubble, and the data label within or to the right of each bubble, corresponds to the total number of conditions or complaints fitting into each combination of body system category and information quantity rating.

Data on information quantity rating was complete for 97.5% (1925/1974) of conditions or complaints listed for guinea pigs (for full details see Table S2, Supplementary Materials). The overall results show that the veterinarians perceived there to be ‘a lot’ of information available for only 4.9% (94/1925) of conditions or complaints they listed. ‘Some’ information was perceived to be available for 26.9% (517/1925) of the conditions or complaints listed, while ‘a little’ information was perceived to be available for 52.3% (1007/1925) of conditions or complaints listed. Only 8.7% (168/1925) of conditions or complaints listed were perceived as having no information available, while a response of ‘don’t know’ was listed for 7.2% (139/1925) of conditions and complaints. It appeared that the body systems categorised more commonly by respondents were often felt to have more information available on them for guinea pigs (Figure 2).

### 3.2. Literature Search

The literature searches in CAB Abstracts returned 7760 citations on rabbits. With categorisation of rabbit citations from every eighth year, this provided 655 citations to categorise, which included a total of 547 relevant citations in English. For guinea pigs, all 1086 citations identified in the literature search were analysed. After removing irrelevant citations and those not in English, there were a total of 620 citations categorised. The number of citations increased per year for both rabbits and guinea pigs [16].

#### 3.2.1. Type of Citation

While the majority of citations described primary research (*n* = 378/547, 69.1% rabbit citations; *n* = 391/620, 63.1% guinea pig citations), a large number of expert opinion citations were also identified (*n* = 169/547, 30.9% rabbit citations; 229/620, 36.9% guinea pig citations). Case studies accounted for 93/378 (24.6%) primary research citations about rabbits and 79/391 (20.2%) primary research citations about guinea pigs. Clinical trials accounted for 130/378 (34.4%) primary research citations about rabbits and 125/391 (32.0%) primary research citations about guinea pigs. Many of these clinical trials involved the use of rabbits (52/130; 40%) or guinea pigs (110/125; 88%) as a model for other species.

#### 3.2.2. Environment

Around half of all citations involved laboratory animals, or were intended for those dealing with animals in a laboratory environment, for both rabbits (262/547; 47.9%) and guinea pigs (318/620; 51.3%) (Table 2). Only 47/547 (8.6%) citations for rabbits and 69/620 (11.1%) for guinea pigs were explicitly described as relating to a pet environment. It was not possible to determine the intended audience for over a third of citations about both rabbits (197/547; 36.0%) and guinea pigs (232/620; 37.4%).

#### 3.2.3. Publication

The rabbit search returned citations in 234 different publications (see Table S3, Supplementary Materials for further details). The publication which provided the most citations in the literature search was ‘Laboratory Animal Science’ (*n* = 18), followed by ‘Exotic DVM’ (*n* = 14) then ‘Laboratory Animals’ (*n* = 11). Seven of the top ten publications were deemed veterinary publications, two laboratory animal publications, and one a human healthcare publication.

The guinea pig search returned citations in 250 publications (see Table S4, Supplementary Materials for further details). The publication which provided the most citations in the literature search was ‘Veterinary Clinics of North America: Exotic animal practice’ (*n* = 23), followed by ‘Antimicrobial Agents and Chemotherapy’ (*n* = 16) then ‘Exotic DVM’ (*n* = 14). Six of the top ten publications were deemed veterinary publications, two laboratory animal publications, and two human healthcare publications.

#### 3.2.4. Body System

In total, 82/547 (15.0%) citations about rabbits and 112/620 (18.1%) citations about guinea pigs could not be categorised by body system, as they did not cover a particular condition or clinical presentation but instead covered a general topic, such as anaesthesia, critical care, or radiography. The remaining 465/547 (85.0%) rabbit citations and 508/620 (81.9%) guinea pig citations were categorised by body system, and are compared with the body system categories derived from the questionnaire in the next section.

### 3.3. Comparison between the Questionnaire Data and the Literature Search

**Figure 3:**
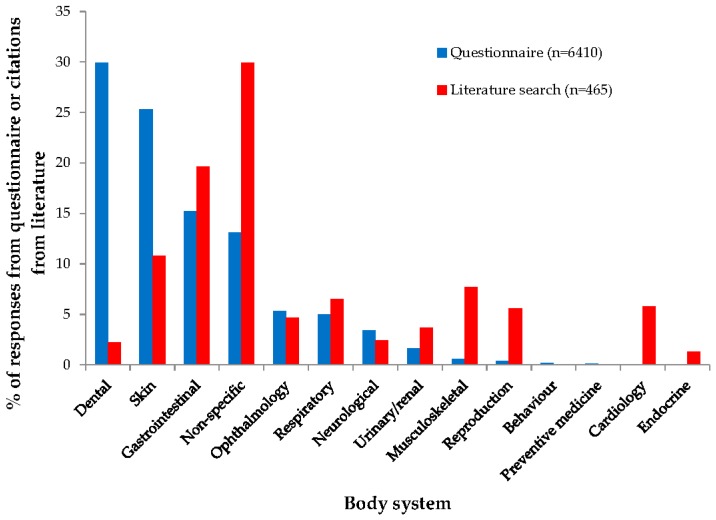
Body system category selected for 6410 rabbit conditions nominated during a survey of UK veterinarians, and 465 rabbit citations identified during a literature search.

There appeared to be a mismatch between the body systems categorised from conditions nominated in the questionnaire and the body systems categorised for citations identified in the literature search about rabbits (Figure 3). While ‘dental’ was the most commonly nominated body system from the questionnaire (*n* = 1918/6410; 29.9%), only a small number of citations identified in the literature review were categorised as ‘dental’ (*n* = 10/465; 2.2%). ‘Skin’ was the second most commonly nominated body system from the questionnaire (*n* = 1619/6410; 25.3%) but accounted for a lower proportion of citations from the literature search (*n* = 50/465; 10.8%).

**Figure 4:**
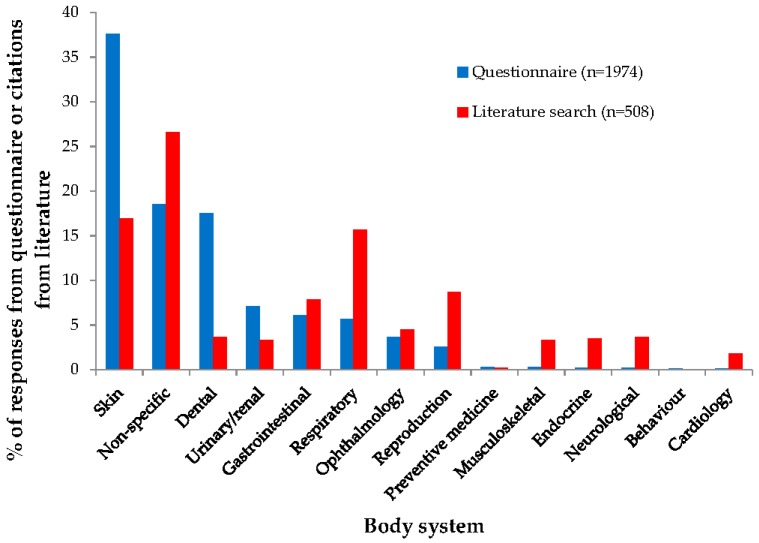
Body system category selected for 1974 guinea pig conditions nominated during a survey of UK veterinarians, and 508 guinea pig citations identified during a literature search.

As with rabbits, there appeared to be a mismatch between the body systems categorised from conditions nominated in the questionnaire and the body systems categorised for citations identified in the literature search for guinea pigs (Figure 4). While ‘skin’ was the most commonly nominated body system from the questionnaire (*n* = 743/1974; 37.6%), only a relatively small proportion of citations identified in the literature review were categorised as ‘skin’ (*n* = 86/508; 16.9%). Similarly, ‘dental’ was the third most commonly nominated body system from the questionnaire (*n* = 346/1974; 17.5%) but only a small number of citations from the literature search were categorised as ‘dental’ (*n* = 19/508; 3.7%).

## 4. Discussion

This is the first time that the published literature has been compared with the nominated caseload from veterinarians focused on rabbits and guinea pigs. While rabbits and guinea pigs appear to be the most common small animal species presented to veterinarians in the UK after cats and dogs, the indexed evidence base identified in this study that is available to veterinarians making decisions about these species appears to be limited, and does not appear to reflect the common conditions seen. Future veterinary research on these species should focus on topics directly relevant to veterinary practice, however efforts should also focus on ensuring the limited existing research-based evidence is easily accessible to the veterinary profession.

Conditions classified into the dental, skin and gastrointestinal categories were the most commonly nominated for rabbits from the veterinarian questionnaire, which is consistent with findings by previous research examining the veterinary caseload [10]. It also closely mirrors findings of two different owner surveys of pet rabbit health [8,11], however, a report by the People’s Dispensary for Sick Animals [1] estimated that only 68% of rabbits are registered with a veterinary practice, suggesting health in the general rabbit population may not accurately reflect the rabbit conditions presented to the veterinarian. Interestingly, veterinarians perceived there to be more information available for dental conditions than for other common conditions, however, the results of the literature review would suggest that this is not the case. Veterinarians frequently access a wide range of peer-reviewed and non-peer reviewed information sources [12], and perhaps because of the scarce indexed evidence in both rabbits and guinea pigs, there is more reliance on information acquired through continuing professional development (CPD), websites and textbooks. While some of the citations were coded as non-specific (e.g., those describing the approach to anorexia) and some of those not coded by body system as they covered a general topic (e.g., critical care) could contain information relevant to dental conditions, only a small number of articles focused on dental disease specifically. It may be that although common, dental issues could be perceived as relatively uncomplicated, or equally complex to manage and, therefore, the interest to grow the research-based evidence on these topics is far less in the veterinary community. This could be the same for skin conditions in both species, as well as common conditions in guinea pigs, such as dental disease, although a recent publication suggests the aetiology of dental disease in guinea pigs is still unclear [17].

Conditions classified into the skin, followed by non-specific (i.e., systemic conditions or non-specific clinical signs, such as inappetance) and dental categories were the most commonly nominated in the veterinary questionnaire for guinea pigs. This differs somewhat from previous findings [9] which identified dental conditions to be most common, followed by skin conditions and ovarian disease. These discrepancies could be down to the different methods used in each study, with one retrospectively examining records [9] while the current study relied on veterinarian recall. Alternatively, the differences seen may reflect a genuine contrast in caseload between a university exotic clinic in the Czech Republic, and the general caseload seen by UK veterinarians. Again veterinarians perceived there to be more information available for the more common conditions in guinea pigs, but results of the literature review suggest that for skin and dental conditions, the amount of literature available does not reflect how common veterinary surgeons perceive these conditions to be.

The amount of information perceived to be available, and the total number of citations identified in the literature search, was lower for guinea pigs than for rabbits, which may reflect the popularity of pet rabbits in the UK compared with guinea pigs [1,2]. The results highlight the challenges facing veterinary surgeons searching for information about rabbits and guinea pigs, with much of the information available not in a veterinary journal, relating to laboratory animals or describing use of rabbits or guinea pigs as a model for disease in other species. While much of this information may still be useful to practising veterinarians, the information may be less directly relevant, as it is not aimed primarily at this audience. Pets inevitably face different disease challenges to animals in a laboratory environment, particularly as there are UK codes of practice which maintain a minimum standard for laboratory animal husbandry and monitoring [18]. In contrast, a recent report highlighted that many pet rabbits in the UK are kept in inadequate housing, fed a suboptimal diet, live alone and do not receive routine veterinary care [1], meaning they may face very different welfare issues. Therefore, the research priorities for laboratory animals will likely be different from those for pet rabbits and guinea pigs. In addition, while many of the diseases for which these species are used as models are also naturally occurring in rabbits and guinea pigs, the conditions studied likely do not reflect the common conditions encountered by veterinarians in first-opinion practice. One potential solution to this problem could be to ensure the limited relevant evidence is easy to access for the veterinary profession. In recent years, there has been a move towards initiatives, such as Best Evidence Topics (BestBETs) for Vets [19], which produces freely-available summaries of the evidence on a focused clinical question relevant to veterinary practice, and published the first summary addressing a clinical question about rabbits in 2016 [20]. Similar initiatives that have also made summaries of the existing evidence available to veterinarians include Critically Appraised Topics (CATs) [21,22] and Knowledge Summaries [23].

In the absence of published research, other sources of evidence, for example textbooks and CPD can be a useful information source for veterinarians seeking evidence to support decision-making. As many of the citations identified in this study were categorised as expert opinion, such evidence may be an alternative source of information for veterinarians treating rabbits and guinea pigs where primary research is not available. While these sources of evidence have traditionally been considered to be weaker forms of evidence than primary research, they could be harnessed in an evidence-based way, for example through the formation of Delphi consensus panels to gather expert opinion in an objective manner [24].

In the longer term, new, high quality, relevant evidence needs to be generated to address the knowledge gaps identified during the study. The mismatches between common conditions nominated by veterinarians and those found in the existing evidence base highlight the need for careful consideration of appropriate topics for future research in these species. There are likely to be fewer funding opportunities for the generation of new evidence involving rabbits or guinea pigs, compared with other companion animals, such as dogs and cats. Therefore, it is important that future research focuses on the topics most important to veterinarians and pet owners involved in the care of rabbits and guinea pigs. Involvement of clinicians, carers, and patients in research prioritisation is well established in human healthcare [25] and these methods have recently been adapted for veterinary medicine, with veterinarians and cat owners participating in setting research priorities for feline chronic kidney disease [26]. Such techniques could be particularly useful for certain topics, such as rabbit dental disease, where there is relatively little published information despite the frequency with which this condition is encountered by practicing veterinarians.

There are various limitations to the data gathered from the veterinarian questionnaire, which have been discussed in more depth previously [4]. In particular, listing of common species and conditions relied on reports by the veterinarian, rather than directly measuring caseload, and it is possible that these things, when compared, are different. This seems unlikely, particularly for conditions affecting rabbits, as both dental and skin diseases have consistently been identified as the most common conditions affecting the pet rabbit population [8,10]. As only papers from every eighth year were examined for rabbits due to time constraints, it is possible that the results from the rabbit literature search do not reflect the existing evidence base as a whole. For example, it is possible that particular conditions are more topical, more frequently funded, or more likely to be researched in certain years, leading to over- or under-representation of certain types of conditions for the years examined. Only one database was searched; however, the choice of database was based on previous research, suggesting that CAB Abstracts gives the best coverage of the veterinary literature [14] and the existence of the veterinary subset function within CAB Abstracts. Additionally, non-English papers were excluded during the searches due to translation time constraints, and it is possible that studies focused on the common conditions affecting rabbits and guinea pigs were excluded. Future work could search other databases and include non-English publications as a next step to ensure all relevant existing evidence has been identified. In addition, critical appraisal of the primary research citations was not conducted, so while the results provide an overview of the quantity of evidence available, they do not give an indication of quality, which is important when considering how the evidence should be applied to veterinary practice. Similarly, veterinarians were only asked about the quantity, and not the quality, of information they perceived to be available, and veterinarians were not given a definition of what constituted ‘information’ during the survey. Despite the limitations, this study goes some way to identifying important gaps that exist in the evidence-base for these frequently encountered species.

## 5. Conclusions

Rabbits and guinea pigs are commonly presented to veterinarians in the UK, yet, in many cases, the evidence available to support clinical decision-making for the most common conditions in these species may be limited. This is the first time this has been examined in the veterinary sector in rabbits and guinea pigs. There are additional challenges facing veterinarians searching for evidence on these species, as much of the available evidence may not be relevant to veterinarians treating pet rabbits and guinea pigs in first opinion practice. Future research should focus on generating new, high-quality research guided by the needs of veterinarians and pet owners.

## Figures and Tables

**Table 1:** Body system categorisation for the conditions or complaints nominated for rabbits (*n* = 6410) and guinea pigs (*n* = 1974) in a survey of UK veterinarians conducting clinical work.

Rabbits	*n*	%	Guinea Pigs	*n*	%
Dental	1918	29.9	Skin	743	37.6
Skin	1619	25.3	Non-specific	365	18.5
Gastrointestinal	974	15.2	Dental	346	17.5
Non-specific	839	13.1	Urinary/renal	140	7.1
Ophthalmology	341	5.3	Gastrointestinal	121	6.1
Respiratory	323	5.0	Respiratory	113	5.7
Neurological	216	3.4	Ophthalmology	74	3.7
Urinary/renal	100	1.6	Reproduction	52	2.6
Musculoskeletal	37	0.6	Preventive medicine	5	0.3
Reproduction	23	0.4	Musculoskeletal	5	0.3
Behaviour	12	0.2	Endocrine	4	0.2
Preventive medicine	8	0.1	Neurological	3	0.2
Cardiology	0	0.0	Behaviour	2	0.1
Endocrinology	0	0.0	Cardiology	1	0.1
Total	6410	100.0		1974	100.0

**Table 2:** Environment from which data were collected, or audience at which the citation was aimed, for 547 rabbit citations and 620 guinea pig citations.

	Rabbit		Guinea Pig	
Environment	*n*	%	*n*	%
Laboratory	262	47.9	318	51.3
Pet	47	8.6	69	11.1
Wild	21	3.8	0	0.0
Farm	20	3.7	1	0.2
Not specified	197	36.0	232	37.4
Total	547	100.0	620	100.0

## Supplementary Materials

The following are available online at www.mdpi.com/2306-7381/4/4/58/s1, Table S1: Information quantity rating for amount of information available on rabbit conditions by body system, as provided by veterinarians in a questionnaire on common conditions, Table S2: Information quantity rating for amount of information available on guinea pig conditions by body system, as provided by veterinarians in a questionnaire on common conditions, Table S3: List of publications containing rabbit citations, Table S4: List of publications containing guinea pig citations.
