# Supplementary material for: Veterinarian Nominated Common Conditions of Rabbits and Guinea Pigs Compared with Published Literature
Source: Vet Sci. 2017 Nov 22;4(4):58. doi: 10.3390/vetsci4040058 (PMC5753638; doi:10.3390/vetsci4040058)
Supplement: Supplementary file 1 [file vetsci-04-00058-s001.pdf]

veterinary sciences

MDPI

Article

Veterinarian Nominated Common Conditions of Rabbits and Guinea Pigs Compared with Published Literature

Natalie J. Robinson [1,*], Emma Grimes [1], Douglas Grindlay [2] and Marnie L. Brennan [1]

[1] Centre for Evidence-Based Veterinary Medicine, School of Veterinary Medicine and Science, The University of Nottingham, Sutton Bonington Campus, Loughborough LE12 5RD, UK; svyeg2@nottingham.ac.uk (E.G.); marnie.brennan@nottingham.ac.uk (M.L.B.)

[2] Centre for Evidence-based Dermatology, The University of Nottingham, Kings Meadow Campus, Nottingham NG7 2NR, UK; douglas.grindlay@nottingham.ac.uk

* Correspondence: natalie.robinson@nottingham.ac.uk; Tel.: +44-115-951-6782

Supplementary Materials:

Body system definitions used to classify conditions or complaints nominated by veterinarians from a UK based survey.

Any disease, injury or set of clinical signs primarily affecting:

Skin: the integumentary system including the external ear canal

Musculoskeletal: the skeleton or skeletal muscles

Neurological: the central or peripheral nerves including conditions of the brain/spinal cord

Ophthalmology: the eyes, eyelids (including third eyelid), tear ducts and other associated structures

Urinary/renal: the upper or lower urinary tract including kidneys, ureters, bladder and urethra

Reproductive: the male or female reproductive tract, including prostate, testes, penis, uterus, ovaries, vagina, vulva and mammary glands, and encompassing problems of pregnancy, parturition and lactation

Gastrointestinal: the gastrointestinal tract, liver, gall bladder and exocrine pancreas

Cardiovascular: the cardiovascular system, including the heart, pericardium and blood vessels

Respiratory: the respiratory system including lungs, trachea and nasal passages

Endocrine: the endocrinological system including disorders of the pituitary, thyroid, adrenal or endocrine pancreas which results in a hormone imbalance

Dental: the teeth, gums or related structures

<u>OR</u>

Non-specific: Any disease, injury or set of clinical signs which cannot be fitted into one of the above categories. This includes systemic diseases which affect multiple body systems, and non-specific clinical signs such as inappetance or pyrexia of unknown origin

Behavioural: Any activity judged to be outside the normal behaviour pattern for animals of that age, where no underlying medical or physiological abnormality can be found

Preventive medicine: Any activity associated with the prevention of a particular disease, injury or clinical sign. Includes vaccination, prevention of parasites, prophylactic neutering, prevention of season, prevention of pregnancy, microchipping and nail clipping (provided this is not done to treat a current nail or pad disorder)

Type of citation

Primary research citation: A citation reporting a research study (of one of the types described by CEVM) in the form of a scientific paper or scientific conference presentation, or reports the synthesis of evidence with clearly defined methods e.g., a systematic review

Expert opinion citation: A citation which does not report a research study or evidence synthesis with clearly defined methods e.g., an editorial, a narrative review, a non-research conference presentation etc.

Environment

The environment/population of animals from which data were gathered or the primary audience for which the article was intended, as defined in the abstract or title of the paper:
Laboratory: Data collected from laboratory rabbits or guinea pigs/in a laboratory environment or primarily aimed at those working with laboratory rabbits or guinea pigs

Pet: Data collected from pets, rabbits or guinea pigs or primarily aimed at those working with pet rabbits or guinea pigs, usually in a veterinary practice setting. Pet in this context is defined as a domestic animal kept for companionship or pleasure.

Wild: Data collected from or primarily aimed at those working with wild, non-domesticated rabbits or guinea pigs

Farm: Data collected from or primarily aimed at those working with farmed rabbits or guinea pigs. Farmed in this context refers to being bred and raised as a production animal.

Not specified: Any article where the environment from which data were collected, or audience at which the article is primarily aimed, is unclear.

Table S1. Information quantity rating for amount of information available on rabbit conditions by body system, as provided by veterinarians in a questionnaire on common conditions.

	**Information Quantity Rating**										
	**None**		**A little**		**Some**		**A lot**		**Don't know**		
	n	**%**	***n***	**%**	***n***	**%**	***n***	**%**	***n***	**%**	**Total**
Dental	12	0.6	327	17.2	912	48	611	32.1	39	2.1	1901
Skin	55	3.4	601	37.6	672	42	194	12.1	77	4.8	1599
Gastrointestinal	19	2	310	32.3	452	47.1	140	14.6	38	4	959
Non-specific	29	3.5	281	34.1	367	44.6	113	13.7	33	4	823
Ophthalmology	7	2.1	132	39.4	144	43	35	10.4	17	5.1	335
Respiratory	4	1.3	99	31	158	49.5	45	14.1	13	4.1	319
Neurological	3	1.4	81	38.2	99	46.7	24	11.3	5	2.4	212
Urinary	6	6.2	47	48.5	31	32	7	7.2	6	6.2	97
Musculoskeletal	6	16.7	18	50	9	25	2	5.6	1	2.8	36
Reproduction	0	0	11	50	5	22.7	5	22.7	1	4.5	22
Behaviour	0	0	6	54.5	3	27.3	0	0	2	18.2	11
Preventive medicine	1	12.5	3	37.5	3	37.5	0	0	1	12.5	8
Renal	0	0	1	50	0	0	0	0	1	50	2
Endocrine	0	0	0	0	0	0	0	0	0	0	0
Cardiology	0	0	0	0	0	0	0	0	0	0	0

Table S2. Information quantity rating for amount of information available on guinea pig conditions by body system, as provided by veterinarians in a questionnaire on common conditions.

	**Information Quantity Rating**										
	**None**		**A little**		**Some**		**A lot**		**Don't know**		
**Body System**	***n***	**%**	***n***	**%**	***n***	**%**	***n***	**%**	***n***	**%**	**Total *n***
Skin	42	5.8	356	49.0	237	32.6	48	6.6	43	5.9	726
Non-specific	48	13.6	196	55.4	70	19.8	6	1.7	34	9.6	354
Dental	17	5.1	179	53.3	101	30.1	24	7.1	15	4.5	336
Urinary	14	10.6	76	57.6	27	20.5	4	3.0	11	8.3	132
Gastrointestinal	13	10.9	59	49.6	32	26.9	4	3.4	11	9.2	119
Respiratory	14	12.5	62	55.4	24	21.4	3	2.7	9	8.0	112
Ophthalmology	12	16.4	31	42.5	17	23.3	1	1.4	12	16.4	73
Reproduction	3	6.0	35	70.0	7	14.0	3	6.0	2	4.0	50
Musculoskeletal	2	40.0	1	20.0	1	20.0	0	0.0	1	20.0	5

Preventive medicine	1	20.0	3	60.0	0	0.0	1	20.0	0	0.0	5
Endocrine	1	25.0	2	50.0	1	25.0	0	0.0	0	0.0	4
Neurological	1	33.3	2	66.7	0	0.0	0	0.0	0	0.0	3
Renal	0	0.0	2	66.7	0	0.0	0	0.0	1	33.3	3
Behaviour	0	0.0	2	100.0	0	0.0	0	0.0	0	0.0	2
Cardiology	0	0.0	1	100.0	0	0.0	0	0.0	0	0.0	1

Table S3. List of publications containing rabbit citations (only journals containing 3 or more rabbit citations are shown).

	**Journal Title**	**Number of Citations**
1	Laboratory Animal Science	18
2	Exotic DVM	14
3	Laboratory Animals	11
4	American Journal of Tropical Medicine and Hygiene	10
5	Indian Veterinary Journal	10
6	Veterinary Record	10
7	World Rabbit Science	10
8	Veterinary Times	9
9	Veterinary Parasitology	8
10	Research in Veterinary Science	7
11	Antimicrobial Agents and Chemotherapy	6
12	Journal of Applied Rabbit Research	6
13	Vaccine	6
14	Veterinary Pathology	6
15	VN Times	6
16	Dissertation Abstracts International, B	5
17	Journal of Veterinary Medicine	5
18	Veterinary Ophthalmology	5
19	Bulletin of the Veterinary Institute in Pulawy	4
20	Indian Journal of Veterinary Pathology	4
21	Infection and immunity	4
22	Irish Veterinary Journal	4
23	Journal of Exotic Pet Medicine	4
24	Journal of Parasitology	4
25	Seminars in Avian and Exotic Pet Medicine	4
26	Veterinary Immunology and Immunopathology	4
27	Acta Tropica	3
28	Asian Pacific Journal of Tropical Medicine	3
29	Australian Journal of Zoology	3
30	British Veterinary Journal	3
31	Contemporary Topics in Laboratory Animal Science	3
32	In Practice	3
33	Indian Journal of Animal Health	3
34	Indian Journal of Animal Sciences	3
35	Journal of Comparative Pathology	3
36	Journal of Veterinary Diagnostic Investigation	3
37	Journal of Veterinary Medical Science	3
38	Journal of Veterinary Parasitology	3
39	Journal of Virology	3
40	Journal of Wildlife Diseases	3
41	Korean Journal of Veterinary Research	3
42	Lucrari Stiintifice—Universitatea de Stiinte Agricole a Banatului Timisoara, Medicina Veterinara	3
43	Theriogenology	3
44	Trakia Journal of Sciences	3
45	Veterinary and Comparative Orthopaedics and Traumatology	3

46	Veterinary Journal	3
47	Veterinary Medicine	3
48	Veterinary Nursing Journal	3

Table S4. List of publications containing guinea pig citations (only journals containing 3 or more guinea pig citations are shown).

	Journal	Number of Citations
1	Veterinary Clinics of North America: Exotic Animal Practice	23
2	Antimicrobial Agents and Chemotherapy	16
3	Exotic DVM	14
4	Laboratory Animal Science	13
5	Veterinary Times	13
6	Vaccine	10
7	Canadian Veterinary Journal	9
8	Laboratory Animals	9
9	Veterinary Ophthalmology	9
10	Veterinary Record	9
11	Journal of the American Veterinary Medical Association	8
12	American Journal of Veterinary Research	6
13	In Practice	6
14	Journal of Exotic Pet Medicine	6
15	Journal of Infectious Diseases	6
16	Veterinary Dermatology	6
17	VN Times	6
18	Journal of Ethnopharmacology	5
19	Journal of Small Animal Practice	5
20	UK Vet: Companion Animal	5
21	Infection and Immunity	4
22	Journal of Antimicrobial Chemotherapy	4
23	Journal of Clinical Microbiology	4
24	Scandinavian Journal of Laboratory Animal Science	4
25	Veterinary Microbiology	4
26	Animal Technology	3
27	Annals of Tropical Medicine and Parasitology	3
28	Australian Veterinary Journal	3
29	Biology of Reproduction	3
30	Clinical and Vaccine Immunology	3
31	Clinical Microbiology and Infection	3
32	Compendium on Continuing Education for the Practicing Veterinarian	3
33	Contemporary Topics in Laboratory Animal Science	3
34	European Journal of Companion Animal Practice	3
35	Indian Veterinary Journal	3
36	International Archives of Allergy and Immunology	3
37	Journal of Endocrinology	3
38	Journal of the American Association for Laboratory Animal Science	3
39	Journal of Veterinary Medical Science	3
40	Journal of Virological Methods	3
41	Medical Mycology	3
42	Mycopathologia	3
43	Revista do Instituto de Medicina Tropical de Sao Paulo	3
44	Veterinary Pathology	3
45	Veterinary Radiology & Ultrasound	3

© 2017 by the author. Licensee MDPI, Basel, Switzerland. This article is an open access article distributed under the terms and conditions of the Creative Commons Attribution (CC BY) license (http://creativecommons.org/licenses/by/4.0/).
